# Women’s cancers in China: a spatio-temporal epidemiology analysis

**DOI:** 10.1186/s12905-021-01260-1

**Published:** 2021-03-20

**Authors:** Rongxin He, Bin Zhu, Jinlin Liu, Ning Zhang, Wei-Hong Zhang, Ying Mao

**Affiliations:** 1 School of Public Policy and Administration, Xi’an Jiaotong University, 28 Xianning West Road, Beilin District, Xi’an, 710049 China; 2 International Centre for Reproductive Health (ICRH), Department of Public Health and Primary Care, Faculty of Medicine and Health Sciences, Ghent University, C. Heymanslaan 10 UZ, 9000 Ghent, Belgium; 3 Research Center for the Belt and Road Health Policy and Health Technology Assessment, Xi’an Jiaotong University, 28 Xianning West Road, Xi’an, 710049 China; 4 School of Public Health and Emergency Management, Southern University of Science and Technology, 1088 Xueyuan Avenue, Shenzhen, 518055 China; 5 School of Public Policy and Administration, Northwestern Polytechnical University, 127 Youyin West Road, Beilin District, Xi’an, 710072 China

**Keywords:** Breast cancer, Cervical cancer, Ovarian cancer, Spatial clusters, Geographical clusters, Moran’s I, Space–time scan, China

## Abstract

**Background:**

Women's cancers, represented by breast and gynecologic cancers, are emerging as a significant threat to women's health, while previous studies paid little attention to the spatial distribution of women's cancers. This study aims to conduct a spatio-temporal epidemiology analysis on breast, cervical and ovarian cancers in China, thus visualizing and comparing their epidemiologic trends and spatio-temporal changing patterns.

**Methods:**

Data on the incidence and mortality of women’s cancers between January 2010 and December 2015 were obtained from the National Cancer Registry Annual Report. Linear tests and bar charts were used to visualize and compare the epidemiologic trends. Two complementary spatial statistics (Moran’s I statistics and Kulldorff’s space–time scan statistics) were adopted to identify the spatial–temporal clusters.

**Results:**

The results showed that the incidence and mortality of breast cancer displayed slow upward trends, while that of cervical cancer increase dramatically, and the mortality of ovarian cancer also showed a fast increasing trend. Significant differences were detected in incidence and mortality of breast, cervical and ovarian cancer across east, central and west China. The average incidence of breast cancer displayed a high-high cluster feature in part of north and east China, and the opposite traits occurred in southwest China. In the meantime, the average incidence and mortality of cervical cancer in central China revealed a high-high cluster feature, and that of ovarian cancer in northern China displayed a high-high cluster feature. Besides, the anomalous clusters were also detected based on the space–time scan statistics.

**Conclusion:**

Regional differences were detected in the distribution of women’s cancers in China. An effective response requires a package of coordinated actions that vary across localities regarding the spatio-temporal epidemics and local conditions.

**Supplementary Information:**

The online version contains supplementary material available at 10.1186/s12905-021-01260-1.

## Background

Due to the differences in physiological structure, women are suffering more from some serious cancers that start in the reproductive system or breast tissue, among which, breast cancer, cervical cancer and ovarian cancer are three common cancers. These cancers result in enormous psychological and economic burdens [1]. Breast cancer is the most common cancer in women. Its established risk factors include overweight, family history, lifestyle and reproductive factors [2, 3]. According to the GLOBOCAN 2018 database [4], there are about 2.1 million newly diagnosed female breast cancer cases in 2018, accounting for almost 1 in 4 cancer cases among women. The disease is the most frequently diagnosed cancer in the vast majority of the countries (154 of 185 countries) and is also the leading cause of cancer death all over the world. Cervical cancer is the most common cancer of the female genital system arising from the cervix. Cervical cancer had 569,847 cases (4th in the world’s female cancer incidence ranking) in 2018 globally and it always caused more casualties in underdeveloped countries. Its prevention requires massive health resources input, i.e., universal screening programs and early treatment [4]. Ovarian cancer is the 8th most common female malignancy cancer (295,414 cases) in the world [4]. Despite its relatively unremarkable incidence, it has the highest mortality in developed countries and the second highest mortality in the developing world among all the gynecologic malignancies [5].

According to the Global Cancer Observatory (GCO) data released by the International Agency for Research on Cancer [6], in 2020, it is estimated that the incidence and mortality rates of breast cancer and ovarian cancer in China are lower than worldwide average and those in Eastern Asia, Western Europe, Northern America countries, higher than those in South-Eastern Asia countries. But the incidence and mortality rates of cervical cancer is significantly higher than that in western countries, which is at the same level as worldwide average and that in the Eastern Asia countries. All the detail data of the incidence and mortality can be found in the Additional file 1: Table S1. As the most populous country in the world, China has about 674.56 million females, which accounted for approximately one-fifth of women all around the world [7]. So it is no doubt that China has a very large population of women with these cancers, Chinese women are impacted more by women’s cancers due to the changes in demographics, reproductive patterns, age structure and lifestyles [2, 8–11].

In the academic world, more and more attention has been paid to the epidemics of women’s cancers in China. As for breast cancer, Fan et al. [12] systematically reviewed the incidence, risk factors and screening programs of breast cancer in China and found a younger age of breast cancer onset in China compared with high-income countries. With the population-based cancer registries’ data, Zeng et al. [13] and Jia et al. [14] estimated the incidence and mortality of female breast cancer in China in 2010 and 2011, respectively. The results indicate that the incidence and mortality of breast cancer were both higher in urban areas than in rural areas. He et al. [15] examined the urban–rural differences in the mortality of breast cancer from 2002 to 2008 and found substantial increases in breast cancer mortality in urban women in China. In the case of cervical cancer, Li et al. [16] investigated the incidence and clinical characteristics of cervical cancer cases based on a nationwide survey of 10,012 cases from 2000 to 2009 and found the increasing incidence in young patients. Shi et al. [17] reviewed the incidence of HPV infection in both China and Mongolia and found a relatively higher incidence in the rural setting. Huang et al. [18] evaluated the long-term temporal trends in the incidence and mortality of cervical cancers in urban Shanghai, China from 1973 to 2012, an upward trend of incidence was found in younger women (age < 60). Zhang et al. [19] investigated the HPV genotype incidence in Chinese women in a 4-year surveillance study (1664 cases). Shi et al. [20] conducted a meta-analysis and estimated the incidence of high-risk HPV infections in women aged 30–54 years in Shanxi to be 17.2%, respectively. Regarding ovarian cancer, Teng et al. [21] reviewed the temporal trends of age-specific incidence of ovarian cancer and summarized its increase in Jiangsu province, which is one of the most developed provincial units in China. Shen and colleagues [11] compared the incidence of malignant and borderline ovarian cancer in pre-menopausal and post-menopausal women in China, and they found that the incidence of ovarian cancer among women before menopause was higher than that of Caucasians. Additional studies can be found to compare the incidence of women’s cancers in the global setting [5, 9, 22].

The previous studies provided plenty of evidence for the worsening epidemic of women’s cancers in China. However, previous studies are either conducted from a temporal perspective or a comparative perspective, but few paid attention to the spatial variations of women’s cancers in China. Females in different areas do not suffer equally from women’s cancers. The incidence of women’s cancers is always heterogeneous across geographic settings, either between countries or within a country [23], which may be attributed to the inequality in health services accessibility, socioeconomic and environmental differences [9]. Understanding the spatial disparities between different geographical units serves as a basis for identifying the populations at high risk and making region-specific prevention and control strategies targeting the high-risk areas.

To fill the research gap, this study aims to review the epidemiologic characteristics and spatial distribution of breast, cervical and ovarian cancers from a spatio-temporal perspective. We believe that the findings will be valuable for making area-targeted prevention interventions, especially the screening programs and critical illness insurance policies.

## Methods

### Data resources

The incidence and mortality data by women’s cancer sites were estimated by the National Cancer Center (NCC) using the data from cancer registries in China. Since 2010, the NCC established and improved cancer registration reporting systems in different regions, achieved the general objective of cancer registration in China. Until 2015, a total of 501 cancer registries submitted data to NCC, 388 of them were included, covering a total of over 320,915,849 population, accounting for 23.35% of the national population. So we collected the province-level year-end incidence and mortality data of breast, cervical and ovarian cancers between 2010 and 2015 from the National Cancer Registry Annual Report, which was published by the NCC [24]. However, Xizang was not included in this research database, due to the data deficiency in most years.

The incidence and mortality data were calculated by the ratio of incident cases of one specific cancer and the number of participants in each provincial unit. Completeness and reliability of submitted data were checked and evaluated by National Cancer Registry Center based on “Guideline for Chinese Cancer Registration” and referring to relevant data quality criteria of "Cancer Incidence in Five Continents Volume 9” by International Association of Cancer Registries. All the original data of the incidence and mortality can be found in the Additional files 2 and 3: Table S2 and S3.

### Spatial statistics

Two complementary spatial statistics (Moran’s I statistics and Kulldorff’s spatial scan statistics) were adopted to detect the spatial clusters (units whose statistics reached the significance level) of the cases of women’s cancers.

#### Moran’s I

Spatial autocorrelation is one kind of spatial statistics, which is used to reveal the spatial structure of regional variable. And the Moran’s I is one of the most common spatial autocorrelation indicators [25–28], which has the unique advantage of analyzing the spatial distribution characteristics of disease cases. There are two detailed indexes of Moran’s I: global and local Moran’s I. Global Moran’s I was used to identifying spatial autocorrelation and detect the spatial distribution pattern of the whole area. And Local Moran’s I was used to examined the local level of spatial autocorrelation and the locations of clusters. The formulas of two indexes of Moran’s I are defined as follows:

$$\mathrm{Global }{\mathrm{Moran}}^{\mathrm{^{\prime}}}\mathrm{I}=\frac{n{\sum }_{i=1}^{n}{\sum }_{j=1}^{n}{SW}_{ij}\left({y}_{i}-\stackrel{-}{y}\right)({y}_{j}-\stackrel{-}{y})}{({\sum }_{i=1}^{n}{\sum }_{j=1}^{n}{SW}_{ij}){\sum }_{i=1}^{n}{({y}_{i}-\stackrel{-}{y})}^{2}}$$

1 $${\mathrm{Local Moran}}^{\mathrm{^{\prime}}}\mathrm{I}=\frac{\left({y}_{i}-\stackrel{-}{y}\right)}{{u}_{0}}{\sum }_{j}{SW}_{ij}\left({y}_{j}-\stackrel{-}{y}\right) {u}_{0}={\sum }_{j}{\left({y}_{i}-\stackrel{-}{y}\right)}^{2}/n$$

$\mathrm{n}$: the number of geographical units (31 provincial units in this study), ${\mathrm{y}}_{\mathrm{i}}$: the incidence or mortality of women’s cancers in geographical unit I, ${\mathrm{y}}_{\mathrm{j}}$: the incidence or mortality of women’s cancers in geographical unit j, $\stackrel{-}{\mathrm{y}}$: the average incidence or mortality of women’s cancers in all the geographical units, ${\mathrm{SW}}_{\mathrm{ij}}:$ spatial-weighted n × n matrix which represents neighboring relations. ${\mathrm{SW}}_{\mathrm{ij}}$= 1 if unit i is adjacent with unit j, and ${\mathrm{SW}}_{\mathrm{ij}}$= 0 otherwise.

Global Moran’s I is an index ranging from − 1 to 1 [29]. When the index was reached 1, the whole spatial distribution displayed the similarity, indicating that the cluster was bounded on other clusters which with similar value [30]. When the index approaching − 1, the overall spatial distribution revealed the dissimilarity, indicating an opposite direction, the cluster was bounded on other clusters which with opposite value. The local Moran’s I detect the spatial autocorrelation in local regions. This study divided local regions by the administrative divisions in China. The cluster results obtained from local Moran’s I were subdivided into four types: High–High cluster (HH), regional units with high value were surrounded by other regional units with high value; High–Low cluster (HL), regional units with high value were surrounded by regional units with low value; Low–Low cluster (LL), regional units with low value were surrounded by other regional units with low value; Low–High cluster (LH), regional units with high value were surrounded by other regional units with low value [31]. The local clusters were visualized using Local Indicator of Spatial Association (LISA) cluster maps [30]. Statistical significance evaluated by using permutation tests with 99,999 replicates and a significance level of 0.05.

#### Kulldorff’s space–time scan statistics

The Kulldorff’s space–time scan statistic is defined by a cylindrical window with a circular geographic base and a height corresponding to time while the height reflects the time period of potential clusters [32]. The first step of space–time scan analysis is to impose a circular window on the map and then it moves in space and time. The window visits each possible geographical location and time period with each possible window size. For this analysis, Poisson based model was used, where the number of events in an area is Poisson distributed according to a known underlying population at risk, the geographic size of the window was limited to half the expected number of cases and the time period was also limited to half the total time period. The difference in the incidence inside and outside the windows was evaluated by the Log-Likelihood Ratio (LLR) as follows [26].

2 $$\mathrm{LLR}=\mathrm{log}\left\{{(\mathrm{C}/\mathrm{n})}^{\mathrm{c}}{\left[(\mathrm{C}-\mathrm{c})/(\mathrm{C}-\mathrm{n})\right]}^{(\mathrm{C}-\mathrm{c})}\right\}$$

C: the total number of cancer cases, $\mathrm{c}$: the number of observed cancer cases inside the space-scan window, n: the number of expected cancer cases inside the space-scan window.

Based on the values of LLR, the space–time scan statistic could identify both the high-risk clusters (the incidence of geographical units within the window is significantly higher than that of units outside of the window) and low-risk clusters (the incidence of geographical units within the window is significantly lower than that of units outside of the window). For either high-risk or low-risk clusters, window with the largest LLR is referred to as the most likely cluster, while others (if any) are known as secondary clusters. Monte Carlo randomization (9999 permutations) was employed to compute the significance of Kulldorff’s spatial scan statistics, with 0.05 being the significance threshold. The maximum radius of the circular base was set at 50% of the total population at risk and the maximum height of the cylinder was set at 50% of the total study period.

### Software tools

The values of Moran’s I were calculated by using the software GeoDa 1.8.61 (the University of Chicago, Chicago, IL, USA). The bar chart were drawn with Microsoft Excel 2016 (Microsoft Corp., Redmond, WA, USA). The chi-square linear by linear association test and seasonal decomposition were conducted in SPSS 20.0 (IBM Inc., Armonk, NY, USA). The space–time scan statistic was measured with the SaTScan 9.5 (Kulldorff and Information Management Services, Inc., Boston, MA, USA). All the maps were drawn and visualized in ArcGIS 10.0 (ESRI Inc., Redlands, CA, USA).

## Results

### Epidemiologic trends

Table 1 shows the incidence and growth rates from 2010 to 2015 in China. For breast cancer, the incidence in all regions showed an increasing tendency. The incidence in the east and west region increased by 12.70% and 18.67%, respectively, while the growth rates displayed a reverse trend in the central region. Except for the east region, the mortality all presented a downward trend. As for cervical cancer, the incidence and mortality were ascending notably, with the growth rate of incidence and mortality in all regions as high as 21.54% and 43.10%, respectively. Regarding ovarian cancer, the incidence did not increase significantly, but the mortality presented an upward trend.

**Table 1:** The growth rates of region-specific incidence and mortality for women’s cancers in China and linear test

	Type	Region	2010 (1/100,000)	2015 (1/100,000)	Between-group difference in time Trend	Growth rates (%)
Incidence	Breast cancer	East	44.73	50.41	5.68	12.70**
		Central	36.83	34.77	− 2.06	− 5.59
		West	25.23	29.94	4.71	18.67*
		All regions	40.69	42.57	1.88	4.62
	Cervical cancer	East	13.36	14.99	1.63	12.20**
		Central	14.18	18.90	4.72	33.29**
		West	11.74	15.41	3.67	31.26**
		All regions	13.37	16.25	2.88	21.54**
	Ovarian cancer	East	7.89	8.22	0.33	4.18*
		Central	6.78	6.70	− 0.08	− 1.18
		West	6.88	7.08	0.20	2.91
		All regions	7.51	7.60	0.09	1.20
Mortality	Breast cancer	East	10.70	11.40	0.70	6.54
		Central	9.94	9.31	− 0.63	− 6.34
		West	8.92	7.93	− 0.99	− 11.10
		All regions	10.32	10.23	− 0.09	− 0.87
	Cervical cancer	East	3.32	4.45	1.13	34.04**
		Central	4.10	6.04	1.94	47.32**
		West	3.72	5.50	1.78	47.85**
		All regions	3.55	5.08	1.53	43.10**
	Ovarian cancer	East	3.67	4.11	0.44	11.99*
		Central	2.83	2.99	0.16	5.65
		West	2.68	2.97	0.29	10.82
		All regions	3.36	3.61	0.25	7.44

Growth rates in parentheses, units that displayed a significant linear trend during the subperiod are in bold. The statistical significance is based on the chi-squared test (* Statistical significance at 10% level; ** Statistical significance at 5% level; *** Statistical significance at 1% level)

**Fig. 1:**
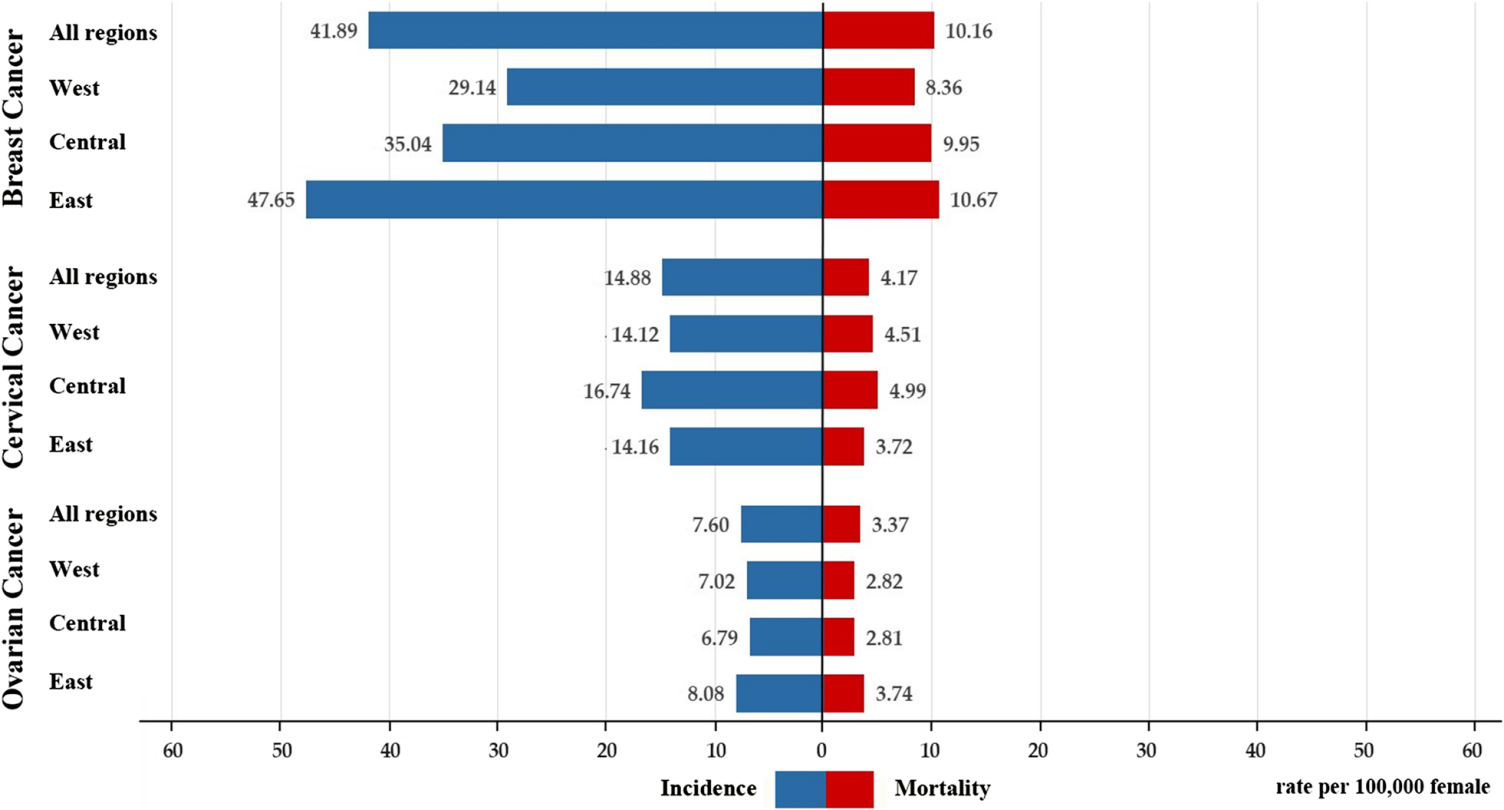
Bar chart of region-specific incidence and mortality for women’s cancers in China (1/100,000)

Figure 1 displays the region-specific average incidence and mortality of breast, cervical and ovarian cancer among women collected during 2010–2015. This study adopted the regional division (east, central and west) in the National Health and Family Planning Statistical Yearbook to classify all provincial units. Overall, geographic differences could be found in the distribution of women’s cancers. The incidence and mortality of breast cancer were highest in the east region (47.65/100,000, 10.67/100,000) and lowest in the west region (29.14/100,000, 8.36/100,000). So there was a significant difference in incidence and mortality of breast cancer across the east, central and west China. In contrast, the mean incidence and mortality of cervical cancer in the central (16.74/100,000, 4.99/100,000) were higher than that of the west (14.12/100,000, 4.51/100,000) and east (14.16/100,000, 3.72/100,000). Besides, the average incidence and mortality of ovarian cancer across regions displayed the same features as that of breast cancer.

### Moran’s I

#### Global Moran’s I

Table 2 summarizes the results of the global Moran’s I for the incidence and mortality of breast, cervical and ovarian cancers. In general, the global Moran’s I for three women cancers were not consistent in the investigated period, with the values fluctuating around 0. The values reached the significance threshold (i.e., α = 0.05) for some years, marked in bold, indicating that the disease cases of women’s cancers were concentrated in certain regions. For instance, the values of the global Moran’s I for the incidence of breast cancer waved in the six years, with the maximum being 0.1613 in 2010 and the minimum being − 0.0461 in 2011 respectively.

**Table 2:** Global spatial autocorrelation analysis and test results^2^

Year	Breast Cancer			Cervical cancer			Ovarian cancer		
	Moran’s I	Z-value	*p*-value	Moran’s I	Z-value	*p*-value	Moran’s I	Z-value	*p*-value
*Incidence*									
2010	**0.1613**	**1.7048**	**0.0488**	− 0.0269	0.0198	0.4813	0.0205	0.4473	0.3081
2011	− 0.0461	− 0.1362	0.4661	− 0.1098	− 0.7365	0.2346	− 0.1042	− 0.6447	0.267
2012	0.1012	1.1691	0.1250	− 0.1578	− 1.1682	0.1160	− 0.0174	0.1159	0.4292
2013	− 0.0095	0.1812	0.4090	0.0419	0.6199	0.2596	− 0.0198	0.0991	0.4486
2014	0.0711	0.895	0.1827	0.1097	1.2386	0.1081	0.071	0.9061	0.1757
2015	0.084	1.011	0.1546	0.0594	0.7798	0.2085	0.0288	0.4661	0.3014
2010–2015	0.0776	0.9604	0.1676	0.0308	0.5217	0.2907	0.0598	0.8063	0.1975
*Mortality*									
2010	0.0039	0.3449	0.3273	− 0.0365	− 0.0747	0.4844	0.0816	0.9925	0.1599
2011	− 0.1315	− 0.9395	0.1698	− 0.098	− 0.6492	0.2601	− 0.1567	− 1.116	0.1275
2012	0.0703	0.9234	0.1754	− 0.0485	− 0.1759	0.4455	0.1113	1.2468	0.1144
2013	0.0452	0.7758	0.1918	0.0593	0.7609	0.2138	− 0.0053	0.1962	0.4001
2014	0.0915	1.1455	0.1229	**0.2053**	**2.0914**	**0.0229**	0.0623	0.8159	0.2012
2015	− 0.0413	− 0.1093	0.4712	0.0803	0.9638	− 0.1659	0.0357	0.5805	0.2647
2010–2015	0.0242	0.5426	0.2814	0.119	1.3405	0.0870	0.0642	0.8297	0.1982

The values marked in bold reached the significance threshold (Statistical significance at 5% level)

#### Local Moran’s I

**Fig. 2:**
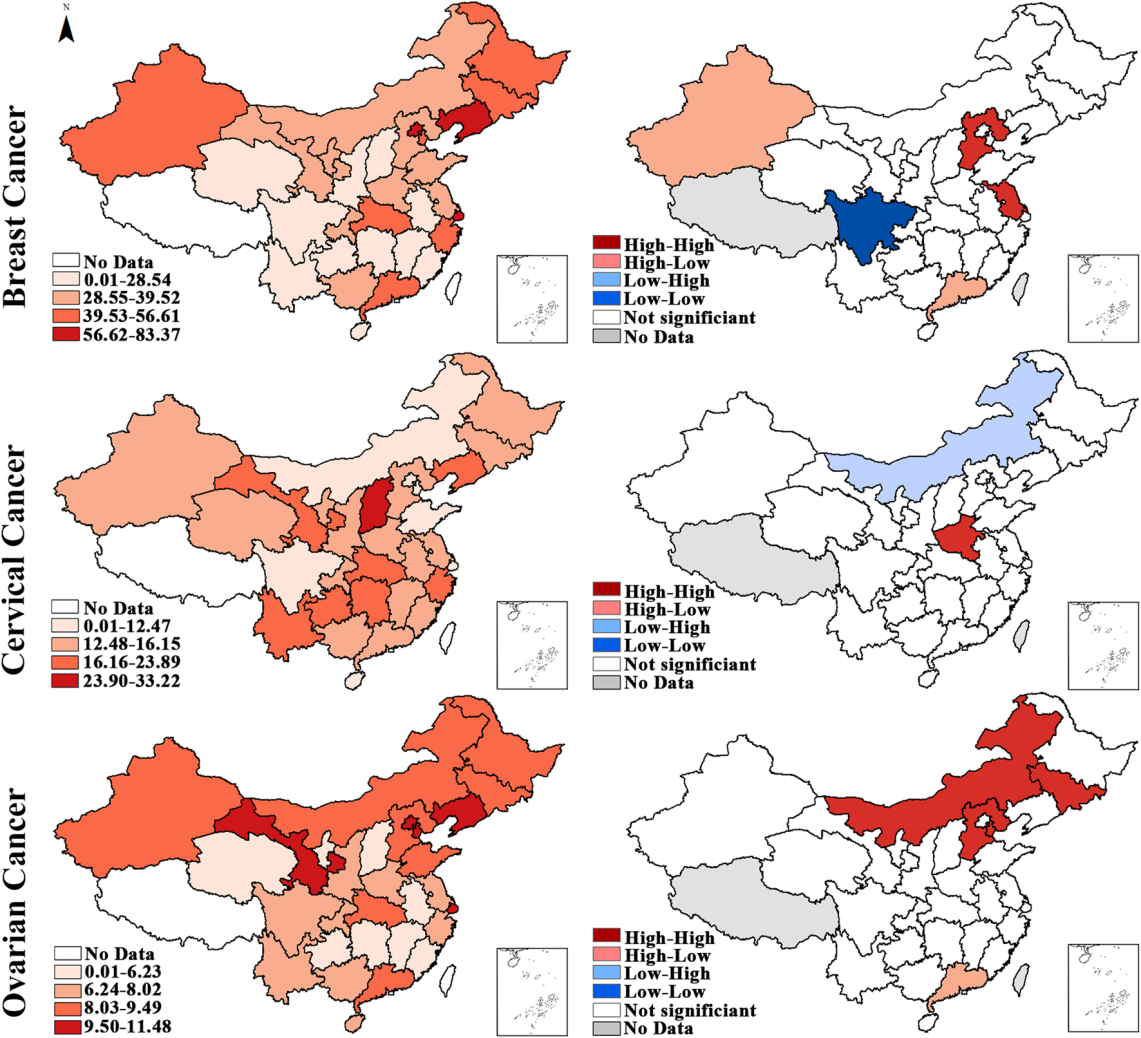
Hierarchical maps (left) and univariate LISA cluster maps (right) of the incidence of breast, cervical and ovarian cancers among women in China. * High-High cluster presents geographical units with high incidence surrounded by geographical units with high incidence, High-Low cluster presents geographical units with high incidence surrounded by geographical units with low incidence, and so on

**Fig. 3:**
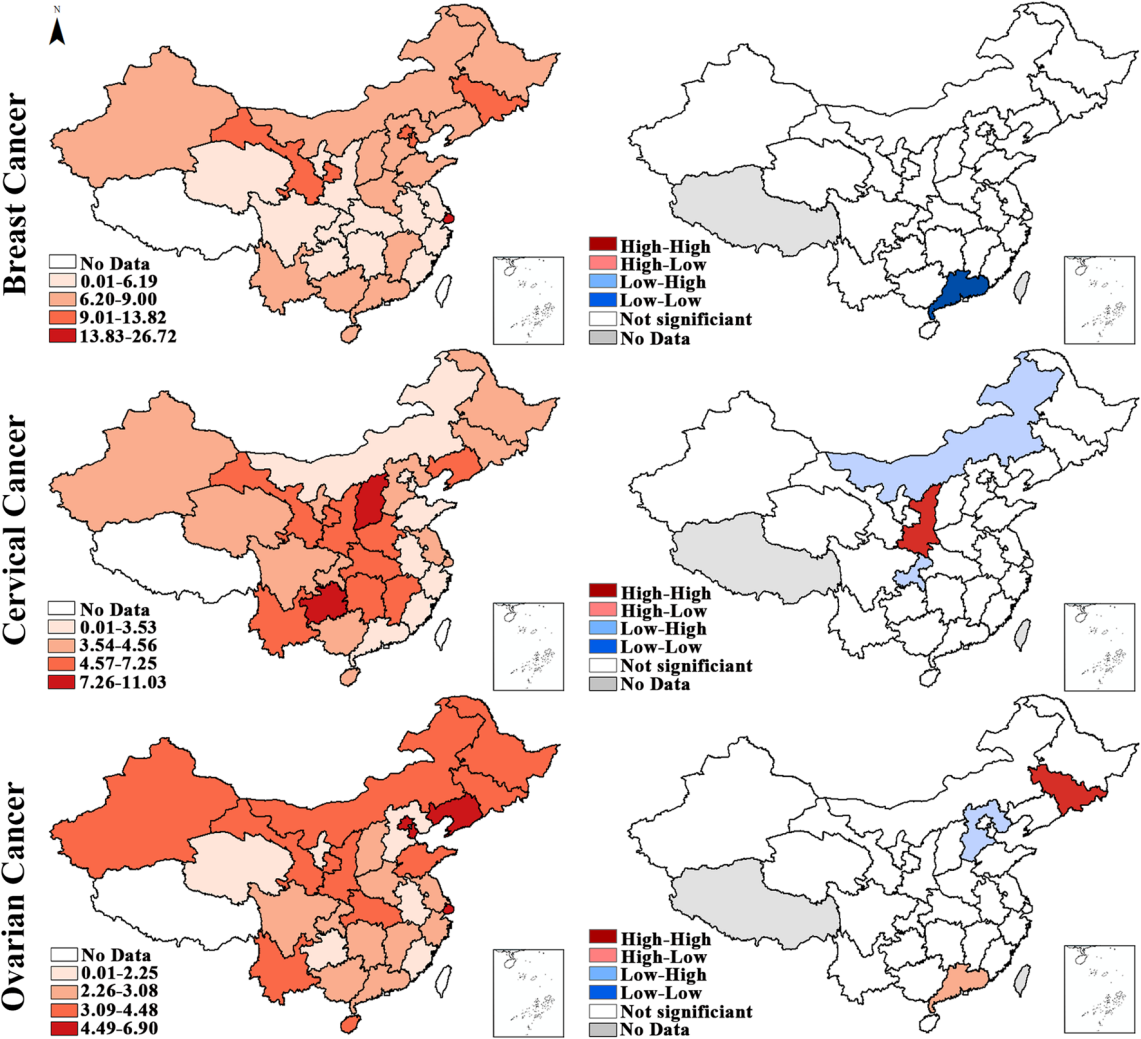
Hierarchical maps (left) and univariate LISA cluster maps (right) of the mortality of breast, cervical and ovarian cancers among women in China. * High-High cluster presents geographical units with high mortality surrounded by geographical units with high mortality, High-Low cluster presents geographical units with high mortality surrounded by geographical units with low mortality, and so on

Figures 2 and 3 show the hierarchical maps and univariate LISA cluster maps for the incidence and mortality of three women's cancers, respectively, demonstrating the detailed spatial distribution and spatial cluster features across neighboring units. The maps have been developed based on the total incidence of breast, cervical and ovarian cancers during 2010–2015.

According to the incidence, for breast cancer, Hebei and Jiangsu displayed the HH cluster feature, indicating the high incidence of breast cancer in them and their neighboring units. Guangdong and Xinjiang displayed the HL cluster feature, which means that the average incidence in its adjacent units is relatively low. Sichuan in southwest China displayed the LL cluster feature, reflecting the relatively low incidence in it and its neighboring units. Regarding cervical cancer, the incidence of Henan and Neimenggu exhibited the HH and LH cluster features, respectively. As for ovarian cancer, the incidence in north China was relatively high, which was evidenced by the significant HH cluster feature in Neimenggu, Jilin, Tianjin and Hebei. Furthermore, the HL cluster features were identified in Guangdong.

According to the mortality, for breast cancer, Guangdong in southeast China displayed the LL cluster feature, indicating the relatively low mortality in this region. Regarding cervical cancer, the mortality of Shannxi exhibited the HH cluster feature. In contrast, the mortality in Chongqing and Neimenggu displayed the LH cluster feature, which indicated that the average mortality in its adjacent units (central China) was relatively high. As for ovarian cancer, the mortality in northeast China was relatively high, which was demonstrated by the significant HH cluster feature in Jilin. HL and LH cluster features were identified in Guangdong and Hebei, respectively.

### Space–time scan analysis

**Fig. 4:**
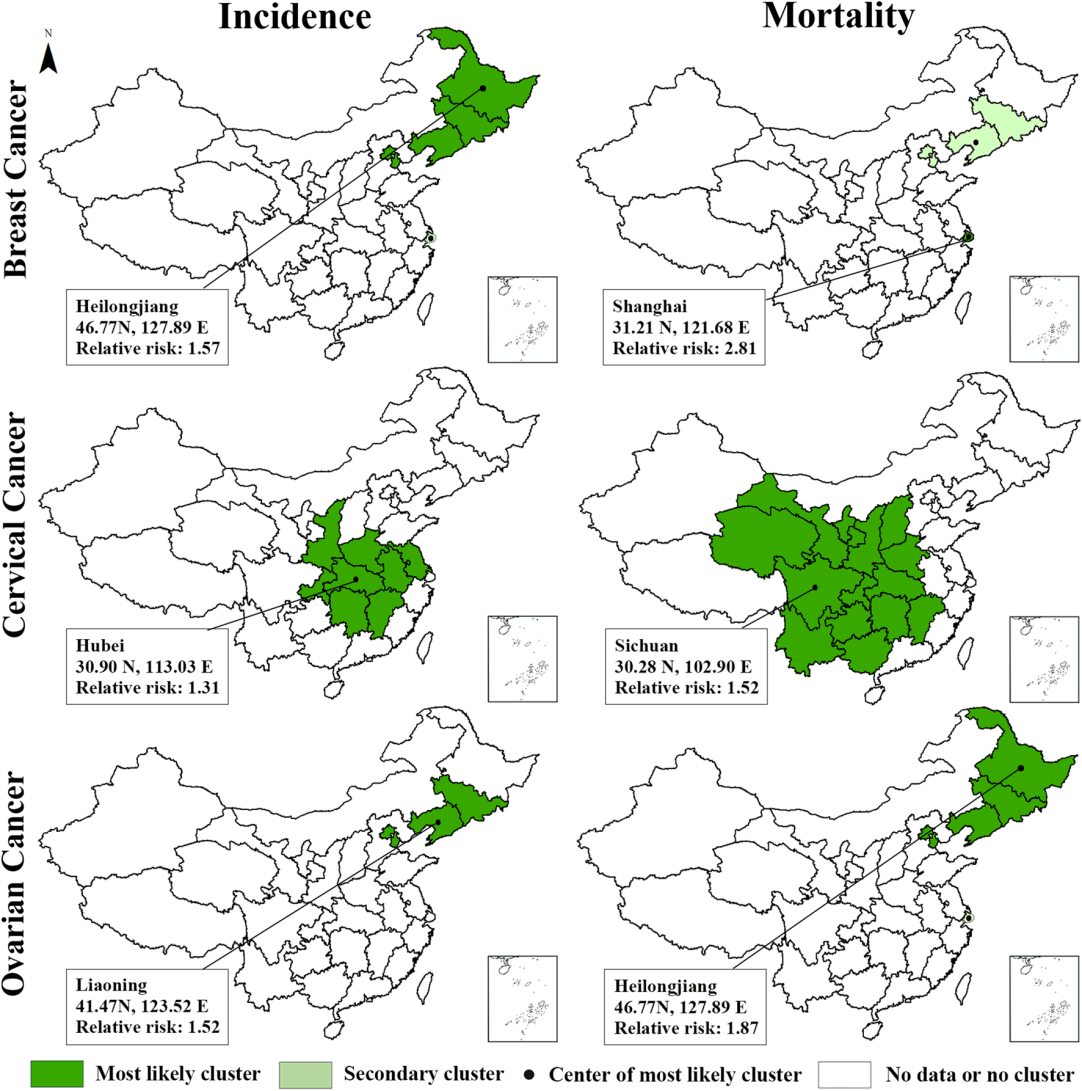
High-risk clusters of the breast, cervical and ovarian cancers among women in China (incidence clusters on the left and mortality clusters on the right)

The results of the space–time scan analysis are shown in Fig. 4, with the spatial clusters of high-risk units of incidence being displayed on the left side and the units of mortality on the right side. The high-risk units of breast cancer and ovarian cancer come with a high degree of consistency. The most likely clusters were concentrated in northeast China, with Heilongjiang, Liaoning and Shanghai being the cluster center. In contrast, the most likely cluster of the high incidence and mortality units of cervical cancer were both concentrated in central China, and the clusters were located in Hubei and Sichuan relatively. The detailed information about the most likely clusters and secondary high-risk clusters of the breast, cervical and ovarian cancers are presented in Table 3.

**Table 3:** The most likely and secondary high-risk clusters of the breast, cervical and ovarian cancers among women in China

Type	Cluster type	Cluster center	Location IDs included	Coordinates	Radius (km)	Number of cases	Expected cases	Annual cases/100,000	Relative risk	LLR	P-value
Incidence of breast cancer	1	Heilongjiang	Heilongjiang, Jilin, Liaoning, Beijing, Tianjin	46.77 N, 127.89 E	1196.92	41,195	27,720.26	61.7	1.57	3212.13	< 0.001
Incidence of breast cancer	2	Shanghai	Shanghai	31.21 N,121.68 E	–	8120	3880.36	86.8	2.13	1788.97	< 0.001
Incidence of cervical cancer	1	Hubei	Hubei, Henan, Hunan, Anhui, Jiangxi, Chongqing, Shaanxi, Jiangsu	30.90 N,113.03 E	757.84	36,177	30,245.00	17.9	1.31	803.67	< 0.001
Incidence of ovarian cancer	1	Liaoning	Liaoning, Jilin, Tianjin, Beijing	41.47 N, 123.52 E	610.40	6649	4592.31	10.9	1.52	450.38	< 0.001
Mortality of breast cancer	1	Shanghai	Shanghai	31.21 N,121.68 E	–	2553	931.11	27.3	2.81	973.23	< 0.001
Mortality of breast cancer	2	Liaoning	Liaoning, Jilin, Tianjin, Beijing	41.47 N, 123.52 E	610.40	8747	6051.71	14.4	1.51	587.29	< 0.001
Mortality of cervical cancer	1	Sichuan	Sichuan, Chongqing, Guizhou, Gansu, Shaanxi, Yunnan, Ningxia, Qinghai, Hunan, Hubei, Guangxi, Henan, Shanxi, Jiangxi	30.27 N,102.90 E	1268.86	7576	5489.85	5.9	1.52	450.31	< 0.001
Mortality of ovarian cancer	1	Heilongjiang	Heilongjiang, Jilin, Liaoning, Beijing, Tianjin	46.77 N, 127.89 E	1196.92	3834	2227.43	5.7	1.87	541.10	< 0.001
Mortality of ovarian cancer	2	Shanghai	Shanghai	31.21 N,121.68 E	–	653	312.25	7.0	2.12	143.65	< 0.001

The radius is reported only when the cluster areas include more than one unit and the criteria for Reporting Secondary Clusters is “no geographical overlap." As for the cluster type, "1" represents "Most likely cluster"; "2" represents "Secondary cluster." The results scanned by elliptic scanning window can be found in the Additional file 4: Table S4

## Discussion

The investigation of cancer epidemics is crucial to understand the situation of women’s cancers in China. However, the existing studies are always conducted from a temporal perspective but paid little attention to their spatial disparities. To supplement the previous studies, this study conducted a spatial–temporal epidemiology analysis of the incidence of breast, cervical and ovarian cancers in China and visualized their trends and spatial changing patterns. We believe that the findings will supplement the existing studies and provide more new evidence for the prevention and surveillance of women’s cancers in China.

Overall, the incidence and mortality of breast cancer displayed slow upward trends, while that of cervical cancer increased dramatically, and the mortality of ovarian cancer also showed a fast increasing tendency. These results echo the previous studies [4, 33–36]. Substantial spatial disparities exist in the spatial distribution of breast, cervical and ovarian cancers, which are evidenced by the bar chart. The incidence and mortality of the three cancers showed distinct spatial distribution characteristics and inconsistent spatial cluster features, which may be attributed to various economic, policy, and geographical factors. In this study, two spatial statistics were used to explore the spatial clusters of cancer cases. Local Moran’s I focuses on the spatial disparities between neighboring units, while the space-scan focuses on the geographical differences at a larger scale as the scan window visits each possible location and plays with all possible sizes. Based on the results, more clusters are detected with the space–time scan statistics than the local Moran’s I statistics, indicating that spatial disparities are more likely to be distributed across different regions (e.g., northeast China and central China) rather than across neighboring units. For women’s cancers, prevention and control are both closely related to population screening. Therefore, we would like to discuss the results of the breast, cervical and ovarian cancers and revisit the current screening strategies and programs.

### Temporal trend

In the first place, the incidence and mortality rates of breast cancer during 2010–2015 did not increase significantly all over China, but in the eastern and western regions. Similar to western women, reproductive and hormonal factors, for example, a long menstrual life (mainly based on early age at menarche and later age at menopause), nulliparity, increased age at first livebirth, and limited breastfeeding—are associated with a modestly increased risk of breast cancer in the Chinese population. The fall in the fertility rate and the rise in obesity rate are also contributed to the increase incidence and mortality rates of breast cancer [12, 37–39]. The growing trend of breast cancer is not the only challenge faced by the Chinese health sector, and there is a consensus that Chinese breast cancer patients are always diagnosed at a younger age compared with the western countries, which may be attributed to the genetics and exposure to risk factors [12, 40, 41]. For this reason, screening for breast cancer is believed to be an effective strategy to achieve early diagnosis and better treatment outcomes in China. However, no uniform guidelines for breast cancer screening exist in China so far, and participation in screening varies widely across age groups and geographical regions [41]. In the western countries, mammography is widely used and understood as the standard imaging for breast cancer screening [42], while it is believed that the experience of western screening strategy cannot be simply copied to China. The reasons are as follows. First, mammography does not always obtain the due effect in China because of the smaller breast size and a higher proportion of dense breasts among Chinese women [42]. Second, the financial cost stands in the way for the target population to access mammography screening due to its broad base. In 2005, a national screening program for breast cancer with both mammography and ultrasound was experimented. But later this program was terminated due to the lack of funds [12]. Therefore, it is suggested that ultrasound may be more useful for Chinese women after evaluating the risk of breast carcinoma for individuals [42]. Besides, numerous studies have applied models to explore the risk factors of women’s cancers based on the Chinese sample, which serves as evidence for predicting the individual breast cancer risk of Chinese women [43–45].

Secondly, a worse epidemic can be observed in the temporal trends of cervical cancer and its comparison with the other two types of cancers reconfirms its severity in China. The incidence and mortality were ascending notably, with the growth rates reached 21.54% and 43.10%, respectively. The rapid growth may be attributed to the high abortion and repeated abortion rate, low population coverage of cervical cancer screening [36, 46]. Fortunately, cervical cancer is a vaccine-preventable disease. Chinese domestic HPV prophylactic vaccine was approved in China in 2016 and its widespread use is highly warranted [47]. Nevertheless, it has not been listed in either the Class A or Class B vaccines of the national immunization program and the vaccine could only protect young females as it does not increase the clearance of established HPV infections [48]. Therefore, vaccinating adolescents at a young age, and screening women from the middle age should be the most effective way to protect Chinese women in the future [49]. In China, the maternal-child system (MCH), which consists of public maternal and child health professional institutions in urban and rural areas, acts as a pivotal part in delivering the organized breast and cervical cancer screening. The Women’s Federations were responsible for organizing women to participate in the screening program. As early as the 1970s, the MCH began to take the gynecological diseases screening and cervical cancer screening as a routine work. However, the inadequate government funding on MCH institutions resulted in the decline in funds for cervical cancer screening. It was not until 2009 that the Chinese government launched the national cervical cancer screening program in rural areas for the first time. Although the screening service in this program was provided free for all target women, the participation rate is quite low even in the more developed eastern China, which may be put down to the poor public health awareness and knowledge of cervical cancer [10]. Besides, the screening program was not followed by appropriate screening training and treatment services, which resulted in low capacities of services providers, poor quality of screening services, and low follow-up rates for positive results [50, 51].

Thirdly, the incidence of ovarian cancer was relatively lower than that of breast and cervical cancer, but its mortality rate has increased significantly during 2010–2015. As a highly lethal disease, it is believed that ovarian cancer has not received due attention compared to the other two cancers. The earlier menarche age, later menopause age, together with the lower numbers of pregnancies resulting from the one-child policy, all contribute to total ovulatory cycles and a higher risk of ovarian cancer in China [18]. In addition, the absence of specific symptoms always results in late diagnosing and a higher risk of recurrence [21]. Up to now, there is no systematic screening program or guidelines for Chinese women.

### Spatial characteristic

By regions, there was an evident difference in incidence and mortality rates of women’s cancer across the east, central and west China. The eastern region has the highest incidence and mortality rates of breast and ovarian cancer in the country. The reasons may be the eastern regions have the lower total fertility rate, higher age at first livebirth or lower levels of physical activity [52, 53]. The incidence and mortality rates of cervical cancer in central China are slightly higher than those in east and west region. And the mortality rate in eastern region lower than those in central and west region. This may be related to the higher technical level of diagnosis and treatment in the eastern region.

By provinces, although there was no significant spatial autocorrelation in women's cancer, but the local level of spatial autocorrelation and the locations of clusters were examined by Local Moran’s I. Regarding to breast cancer, significant differences between the west or central China and the east was evidenced by the analysis, with the latter at a higher risk for breast cancer. As the results we mentioned above, the incidence and mortality rates of breast cancer in eastern region were higher than that in western region. Therefore, the HH clusters and HL clusters were observed in the eastern region, which are all economically developed areas, including Beijing-Tianjin-Hebei region, Yangtze River Delta region (Shanghai, Jiangsu, Zhejiang province) and Pearl River Delta region (Guangdong province). According to the existing research, the main reasons for this phenomenon include high work pressure, increased age at first livebirth, and limited breastfeeding in developed areas [37, 54]. For example, the wealthy eastern coastal regions have the lowest total fertility rate. In urban Shanghai the total fertility rate is the lowest of any city in the world (0.81 per 1000 population in 2010), far lower than in most industrialized countries [12]. The bad living habits and psychological state formed by the urban lifestyle and the obesity caused by unhealthy diet [55, 56] also contributed to the HH clusters of breast cancer in the eastern coastal regions. As to cervical cancer, the hotspots were concentrated in central China, and almost all the cold spots were concentrated in coastal areas, which may be attributed to the more effective screening strategy and coverage and other protective factors of cervical cancer (high education level, high income, late age of first sexual behavior, older age of first pregnancy, older age of first birth, and good sexual hygiene) in developed areas [57, 58]. It is noteworthy that the hotspots of ovarian cancer cases are agminated on the map, with the provincial units in the northeast areas being identified as high-risk cluster areas. At present, there is no consistent conclusion about the risk factors of ovarian cancer. But some studies have shown that the incidence of ovarian cancer is related to dietary habits. High-calorie diet and high body-mass index is a risk factor for ovarian cancer [59–61]. On the contrary, the risk of ovarian cancer is reduced in people who eat more vegetarians such as vegetables and fruits [62]. The residents in northern China, including herdsmen in Inner Mongolia, have a meat-oriented or high-calorie diet due to the cold weather [60]. Whether this kind of diet leads to a higher risk of ovarian cancer remains to be further studied.

To summarize, the Chinese health sector is faced with the increasing challenges of breast, cervical and ovarian cancers, while the existing screening programs have not fully met the expectations. Under present conditions, the screening strategy should be differentiated based on the status quo and the cost-effectiveness of various screening methods in different areas. For instance, population screening after risk evaluation and more cost-effective screening methods could be applied to different situations based on the spatio-temporal epidemiology and fund availability.

This study represents one of the first attempts of the application of spatial statistics on the cancer case distribution in the Chinese context, Moran’s I statistic was mostly applied to detect the clusters of infectious disease cases in the space, thus this study may bear limitations. This study was unable to obtain more detailed results for the municipal units or county unit in China. The sample size in some undeveloped provinces is relatively small and data of a few provinces were missing in individual years. And the incidence is not age-standardized as the age composition of the participants is not clear. Besides, the spatial analysis was conducted at the provincial level due to data accessibility and only the spatial matrix based on sharing borders was used in this study.

## Conclusions

Women's cancers are emerging as a significant threat to women's health in China, with the incidence and mortality showing an upward trend. Significant spatial disparities exist in the epidemics of breast, cervical and ovarian cancers, with women in some geographical units at relatively higher risk. The government is supposed to adopt all possible means, including vaccines, screening, follow-up treatment services and financial risk protection, to protect the Chinese women. More importantly, area-targeted screening and follow-up services are suggested to reduce the spatial disparities in cancer epidemiology and access to health services.

## Supplementary information

**Additional file 1: Table S1**. The incidence and mortality of breast, cervical and ovarian cancer in different regions of the world (unit:1/100,000).

**Additional file 2: Table S2**. The incidence and mortality of breast, cervical and ovarian cancer in each provincial unit from 2010 to 2015 (unit:1/100,000).

**Additional file 3: Table S3**. The number of cases and deaths of breast, cervical and ovarian cancer in each provincial unit from 2010 to 2015.

**Additional file 4: Table S4**. The high-risk clusters of the breast, cervical and ovarian cancers among women in China (elliptic scanning window).

## Data Availability

All data generated or analysed during this study are included in this published article and its supplementary information files.

## Abbreviations

WHO: World Health Organization
HPV: Human papillomavirus
NCC: National Cancer Center
LISA: Local Indicators of Spatial Association
HH: High–high
HL: High–low
LL: Low–low
LH: Low–high
LLR: Log-likelihood ratio
MCH: Maternal-child system
