# Supplementary material for: Women’s cancers in China: a spatio-temporal epidemiology analysis
Source: BMC Womens Health. 2021 Mar 20;21:116. doi: 10.1186/s12905-021-01260-1 (PMC7981806; doi:10.1186/s12905-021-01260-1)
Supplement: Supplementary file 4 — Additional file 4: Table S4. The high-risk clusters of the breast, cervical and ovarian cancers among women in China (elliptic scanning window). [file 12905_2021_1260_MOESM4_ESM.docx]

**Table S4.** The high-risk clusters of the breast, cervical and ovarian cancers among women in China (elliptic scanning window)

| **Type** | **Cluster center** | **Location IDs included** | **Coordinates** | **Number of cases** | **Expected cases** | **Relative risk** | **LLR** | **P-value** |
| --- | --- | --- | --- | --- | --- | --- | --- | --- |
| Incidence of Breast Cancer | Heilongjiang | Heilongjiang, Jilin, Liaoning, Tianjin, Beijing | 46.77N, 127.89E | 41195 | 24881.86 | 1.77 | 4990.92 | <0.001 |
|  | Fujian | Fujian, Zhejiang, Jiangxi, Guangdong, Shanghai | 26.00N, 118.03E | 26569 | 17469.63 | 1.58 | 2200.46 | <0.001 |
|  | Hubei | Hubei, Hunan, Shandong | 30.90N, 113.03E | 21154 | 13962.47 | 1.56 | 1695.40 | <0.001 |
| Incidence of Cervical Cancer | Guizhou | Guizhou, Chongqing, Hunan, Guangxi, Yunnan, Sichuan, Hubei, Shaanxi | 26.69N, 106.61 E | 11146 | 5619.03 | 2.11 | 2270.89 | <0.001 |
|  | Anhui | Anhui, Zhejiang, Henan, Shanxi | 32.01N, 117.19E | 10244 | 6713.93 | 1.59 | 865.19 | <0.001 |
|  | Jiangsu | Jiangsu | 32.47N, 119.97E | 10317 | 8004.87 | 1.32 | 334.79 | <0.001 |
|  | Heilongjiang | Heilongjiang, Jilin, Liaoning | 46.77N, 127.89E | 4282 | 2880.44 | 1.51 | 306.23 | <0.001 |
| Incidence of Ovarian Cancer | Heilongjiang | Heilongjiang, Jilin, Liaoning, Tianjin, Beijing | 46.77N, 127.89E | 7160 | 4529.37 | 1.68 | 724.28 | <0.001 |
|  | Guizhou | Guizhou, Guangxi, Chongqing, Sichuan, Hunan, Guangdong, Yunnan, Hainan, Shaanx | 26.69N, 106.61 E | 4872 | 3262.05 | 1.55 | 357.11 | <0.001 |
|  | Henan | Henan, Hubei, Shandong | 33.80N, 113.9 E | 4712 | 3343.81 | 1.45 | 267.93 | <0.001 |
|  | Shanghai | Shanghai | 31.21N,121.68E | 1000 | 707.58 | 1.42 | 54.34 | <0.001 |
| Mortality of Breast Cancer | Jilin | Jilin, Heilongjiang, Liaoning, Shandong, Shanghai | 43.50N, 126.45E | 10712 | 6389.47 | 1.81 | 1370.36 | <0.001 |
|  | Shanxi | Shanxi, Hebei, Tianjin, Beijing | 37.70N, 112.38E | 6990 | 4733.62 | 1.53 | 509.62 | <0.001 |
|  | Hubei | Hubei, Henan, Jiangxi, Hunan, Shaanxi | 30.90N, 113.03E | 5549 | 3867.41 | 1.47 | 344.37 | <0.001 |
| Mortality of Cervical Cancer | Sichuan | Sichuan, Chongqing, Guizhou, Gansu, Yunnan, Shaanxi, Ningxia, Qinghai, Hunan, Guangxi, Hubei | 30.27N,102.90E | 3994 | 1820.06 | 2.39 | 1055.17 | <0.001 |
|  | Jilin | Jilin, Heilongjiang, Liaoning, Shandong | 43.50N, 126.45E | 2383 | 1561.86 | 1.57 | 198.15 | <0.001 |
|  | Anhui | Anhui, Henan | 32.01N, 117.19E | 2021 | 1314.74 | 1.58 | 171.84 | <0.001 |
|  | Hubei | Hebei, Shanxi | 30.90N, 113.03E | 907 | 468.53 | 1.97 | 164.06 | <0.001 |
|  | Jiangsu | Jiangsu, Jiangxi | 32.47N, 119.97E | 2512 | 1680.35 | 1.54 | 191.27 | <0.001 |
| Mortality of Ovarian Cancer | Heilongjiang | Heilongjiang, Jilin, Liaoning, Tianjin, Beijing | 46.77N, 127.89E | 3834 | 1999.48 | 2.11 | 746.37 | <0.001 |
|  | Shanghai | Shanghai | 31.21N,121.68E | 653 | 312.36 | 2.12 | 143.53 | <0.001 |
|  | Guizhou | Guizhou, Chongqing, Hunan, Guangxi, Yunnan, Sichuan, Hubei, Shaanxi | 26.69N, 106.61 E | 1819 | 1251.72 | 1.49 | 120.26 | <0.001 |
